# Study on the Influence of Nickel Plating on the Structure and Properties of Aluminum/Steel Bimetallic Bonding

**DOI:** 10.3390/ma18091898

**Published:** 2025-04-22

**Authors:** Yufei Zhang, Guowei Zhang, Mingjie Wang, Hong Xu

**Affiliations:** 1 School of Materials and Environment, Shanxi Jinzhong Institute of Technology, Jinzhong 030600, China; zyfaihhn@126.com; 2 School of Materials Science and Engineering, North University of China, Taiyuan 030051, China; 3 School of Intelligent Manufacturing, Huanghuai University, Zhumadian 463000, China; 20212259@huanghuai.edu.cn

**Keywords:** Al/steel bimetal, metallurgical combination, compound casting, mechanical property, microstructure

## Abstract

Aluminum/steel bimetal combines the advantages of aluminum alloy and steel, greatly leveraging the value of various industrial fields, especially in improving engine performance and fuel economy. However, it is very difficult to prepare products with good interface bonding strength. The fundamental issue stems from the presence of an excessively thick interface layer and brittle intermetallic compounds. Therefore, this study employed a 50 μm-thick Ni interlayer to control the interface layer thickness, thereby enhancing the Al/steel interfacial bonding strength. A systematic investigation was conducted on the effects of hot dip duration on the interfacial microstructure and mechanical properties of Al/steel bimetal. The influence of hot dip duration on the microstructure and mechanical properties of aluminum/steel bimetal interface was systematically studied. The results show that the 50 μm Ni intermediate layer was used to effectively control the transition layer thickness and improve the interfacial bonding strength of aluminum steel. The thickness of the interface layer gradually increases with the increase in the hot-immersion time. The thickness of the interface layer composed of the two phases of τ_1_-Al_2_Fe_3_Si_3_ and FeAl_3_ on the steel side increases first and then decreases, while the interface layer composed of the two phases of τ_5_-Al_8_Fe_2_Si and Fe_2_Al_5_ on the aluminum side decreases first and then increases. When the hot dip time is 240 s, the shear strength of Al/steel bimetal with 50 μm Ni interlayer showed 75% enhancement compared to Ni-free counterparts.

## 1. Introduction

At present, single-component materials or alloys struggle to meet the comprehensive performance demands of modern industrial production, driving an urgent need for innovative processing technologies [1]. Supported by advanced technological capabilities, the value of bimetal composites has been progressively unveiled, offering solutions to the inherent limitations of monolithic metals. For instance, dual-metal core multilayer boards (MLBs) demonstrate significant advantages over traditional heat sinks and centralized single-metal core MLBs, effectively addressing both thermal dissipation and coefficient of thermal expansion (CTE) mismatch issues in high speed electronic packaging for spacecraft applications [2]. In energy storage innovation, Xu et al. [3] designed a dual-metal oxide-coated NCM811 cathode for lithium-ion batteries, achieving enhanced electronic/ionic conductivity (>10^−3^ s/cm), improved volumetric modulus, and exceptional interfacial stability under high-voltage cycling. The automotive industry’s push toward lightweighting necessitates materials that balance strength with low density. Al/steel bimetal composites emerge as a promising candidate, offering superior strength-to-weight ratios (>30 MPa·m^3^/kg) and design flexibility for structural components. Beyond automotive applications, their high-temperature resistance and corrosion resilience have garnered attention in chemical processing industries. However, fabricating Al/steel bimetal with robust metallurgical interfaces remains a critical challenge [4,5,6]. The stark physical disparities between aluminum and steel—including thermal expansion coefficients, solid solubility limits, and lattice mismatches—create significant interfacial incompatibilities, constraining their composite applications [7]. Furthermore, thick transition layers dominated by brittle Al-Fe intermetallic compounds (IMCs) such as FeAl_3_ and Fe_2_Al_5_ drastically degrade joint strength, severely limiting the industrial adoption of Al/steel bimetal [8,9]. Consequently, suppressing or eliminating these deleterious IMCs has become a focal research area in bimetal development.

Recent studies have demonstrated that introducing interlayers between molten aluminum and solid steel can significantly enhance the bonding strength of Al/steel bimetal composites [8]. To date, various interlayer materials have been explored, including Cr [10], Ag [11], Zn [12,13], Fe [14], Ni [14,15], high-entropy alloys [16], and Cu [17]. Zhang et al. [10] pioneered the use of a 5 μm electroplated Cr interlayer in Al/steel bimetal fabrication via composite casting, achieving a shear strength of 115 MPa. Feng et al. [11] developed Al/steel bimetal castings using a pre-electroplated Ag layer combined with hot-dipped Al-Si alloy casting. The as-cast interfacial transition layer thickness ranged from 7 to 18 μm, with interfacial shear strength reaching 50–84 MPa. Wang et al. [13] investigated the effects of hot dip-galvanized interlayers and process parameters on interfacial properties. Shear strength initially decreased then increased with rising hot dip temperature, peaking at 35.24 MPa under 600 °C. Chen et al. [14] evaluated Fe/Ni pre-plating and post-plating over-aging treatments in Armco process-based continuous hot dip aluminizing. Ni pre-plating enhanced aluminum melt wettability and reduced intermetallic layer thickness. Notably, Ziming B. et al. [15] observed that increasing holding time (up to 15 min) thickened the Ni interlayer in Al/Ni-coated steel bimetal, achieving a maximum shear strength of 13.4 MPa. Despite preliminary studies on Ni interlayers, limited research exists on the role of Ni interlayers in Al/steel bimetal casting dynamics and the impact of hot-dipping duration on Al/Ni-coated steel interfacial evolution. Therefore, this study systematically investigates the mechanistic role of Ni interlayers during Al/steel bimetal solidification and time-dependent interfacial evolution (microstructure-property correlations) in Al/Ni-coated steel under varying hot-dipping durations (30–300 s).

## 2. Experimental Procedures

### 2.1. Experimental Materials

The substrate material selected was low-carbon steel Q235B (Grade equivalent to ASTM A36) [18], supplied by Taiyuan Iron & Steel (Group) Co., Ltd., Taiyuan, China (TISCO). The casting alloy ZL114 (equivalent to ASTM A356), a hypoeutectic Al-Si alloy [19], was procured from Aluminum Corporation of China Limited, Beijing, China (Chalco). Detailed chemical compositions of both materials are provided in Table 1 (steel) and Table 2 (aluminum). A nickel–phosphorus (Ni-P) alloy coating was deposited onto the steel substrate surface via autocatalytic reaction using an industrial-grade nickel plating solution. The bath composition adhered to the ISO 4527 standard, with controlled phosphorus content of 8–12 at% to optimize interfacial compatibility during subsequent aluminum infiltration [20].

### 2.2. Preparation of Al/Steel Bimetal with Ni Interlayer

**Figure 1:**
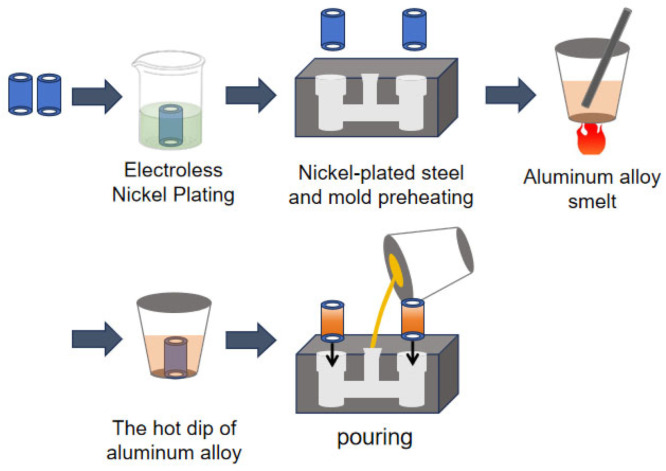
Experimental flow chart.

First, the columnar steel pipe with an outer diameter of 41.5 mm, an inner diameter of 2.5 mm, and a height of 60 mm was sandblasted to remove impurities and oxide layers and improve surface roughness. Soak the cylindrical steel tube in alkaline detergent (NaOH 5% + Na_3_PO_4_ 3% @ 65 °C) for 300 s and ultrasonically rinse in deionized water for 180 s. Post-blast treatment is a dry compressed air purge (0.2 MPa, ISO 8573-1 Class 2) [21]. Immerse in 10% HNO_3_ solution (25 °C) for 120 s and rinse with ethanol (99.7%). Industrial solvents are used to reduce Ni ions to metal Ni and deposit them on the steel surface through chemical action. The process parameters are as follows: plating solution temperature is 90 °C, plating solution pH value is 4.5, and deposition time is 2.5 h. Finally, the plated parts are washed with ionic water and centrifuged and dehydrated. Before casting, the nickel-plated steel was cleaned with an ultrasonic cleaning machine and placed in a 300 °C heating furnace for 5 min. During the composite process, the nickel-plated steel was immersed in 730 °C aluminum melt for hot-dip plating. Then, the nickel-plated steel is placed in the preheated mold, and finally the pure aluminum alloy at 710 °C is poured into the mold to complete the sample preparation of the nickel-plated steel/aluminum bimetal. The experimental protocol was executed following the specified process parameters. A controlled variable approach ensured identical experimental conditions, with hot-dipping duration as the sole manipulated parameter to produce Ni-coated steel/Al specimens across varying immersion times. To minimize experimental variability, three specimens were fabricated for each hot-dipping duration group (30–300 s) and the arithmetic mean of triplicate measurements was adopted as the representative value for each parameter set. The specific experimental process is shown in Figure 1.

### 2.3. Microstructural Characterizations

The morphology of the steel after electroless nickel plating was observed by a scanning electron microscope, the microstructure of the nickel-plated steel/aluminum bimetallic spectroscopy was observed using an SU5000 (Sumitomo Metal Industries, Osaka, Japan) field emission scanning electron microscope, SEM Technologies, Westboro, MA, USA (SEM), and the chemical composition of the interface layer was analyzed using an energy dispersion X-ray energy spectrometer, Thermo Fisher Scientific, Waltham, MA, USA (EDS).

### 2.4. Mechanical Properties

**Figure 2:**
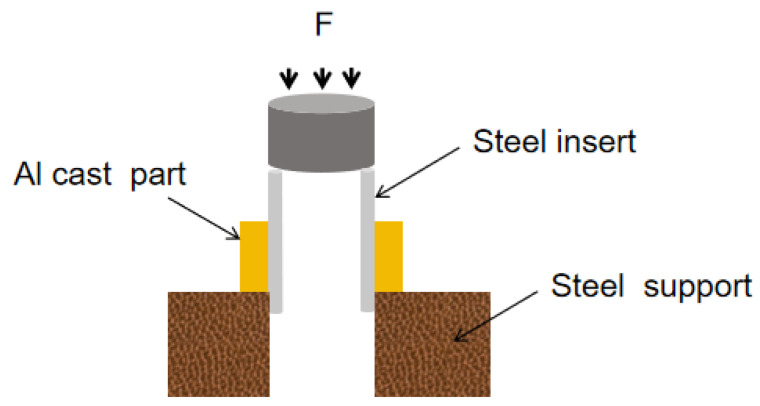
Cutting diagram.

At a displacement rate of 1 mm/min, a universal tester was used to conduct a roll-out test on the interface binding performance of the nickel-plated steel/aluminum bimetallic specimen. Finally, the average value of the three samples was taken as the test result to reduce the error. The cutting diagram is shown in Figure 2.

## 3. Results and Discussion

### 3.1. Nickel-Plated Coating

**Figure 3:**
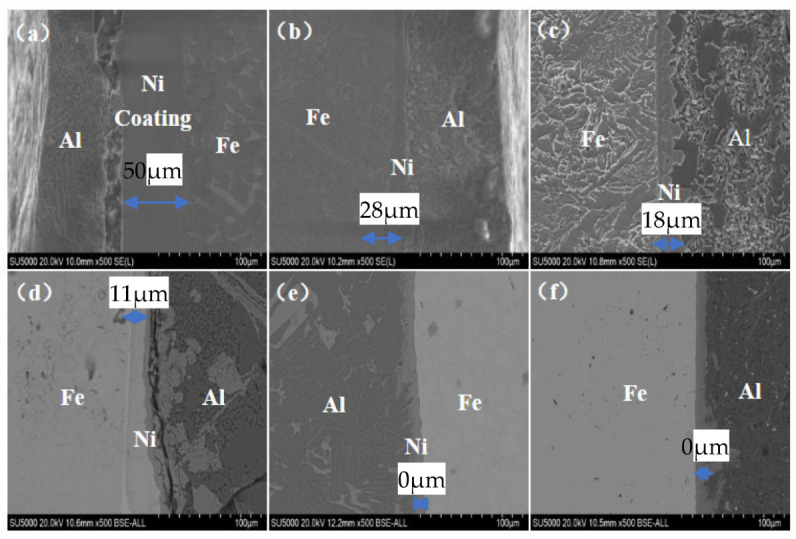
SEM microstructure of nickel-plated steel/Al bimetals in different hot dip times: (**a**) 30 s; (**b**) 60 s; (**c**) 120 s; (**d**) 180 s; (**e**) 240 s; (**f**) 300 s.

Figure 3 shows the microstructure changes of Ni coatings under different hot immersion times. The time for hot-dip aluminum-plated alloy melt is 30, 60, 120, 180, 240, and 300 s, respectively. It can be seen from Figure 3a that after 30 s of hot dip, the solid Ni dissolves in aluminum alloy non-uniformly. It can be seen from Figure 3b that after 60 s of hot dip, part of Ni dissolves, but most of it has not begun to dissolve, indicating that there are certain conditions for Al/Ni diffusion to occur. As can be seen from Figure 3c–f, the Ni layer becomes significantly thinner, resulting in needle-like metal compounds, and gradually grows into the molten aluminum liquid.

### 3.2. Microstructure

**Figure 4:**
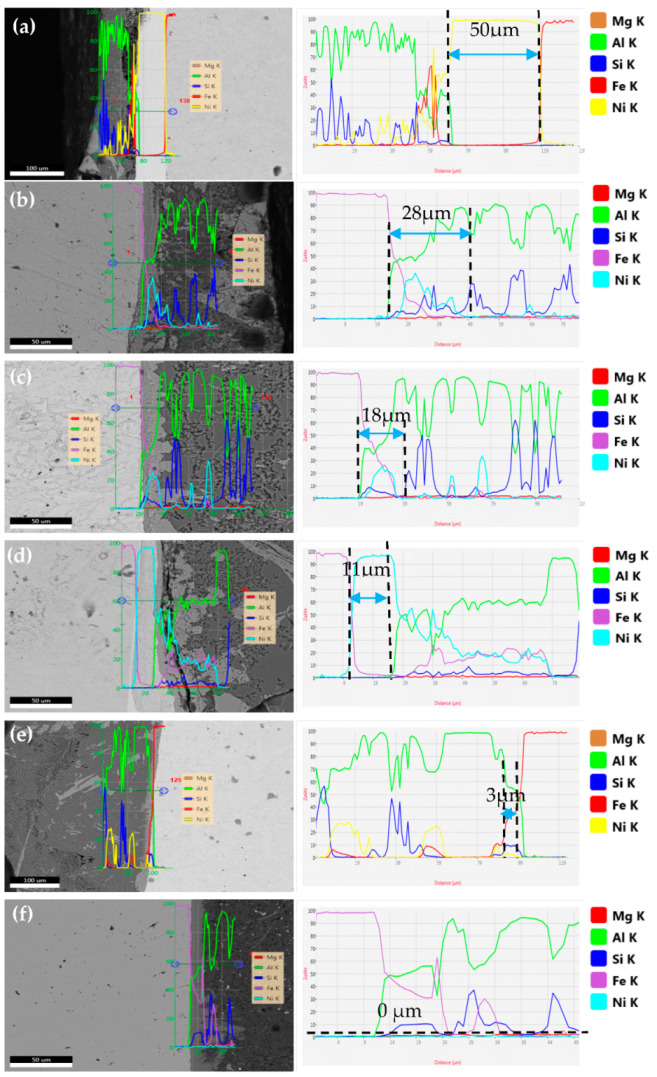
Line scan images of nickel-plated steel/Al bimetals at different hot dip times: (**a**) 30 s; (**b**) 60 s; (**c**) 120 s; (**d**) 180 s; (**e**) 240 s; (**f**) 300 s.

Figure 4 shows the microstructure and line-scan images of the SEM interface of nickel-plated steel/aluminum bimetals of different hot dip times.

**Figure 5:**
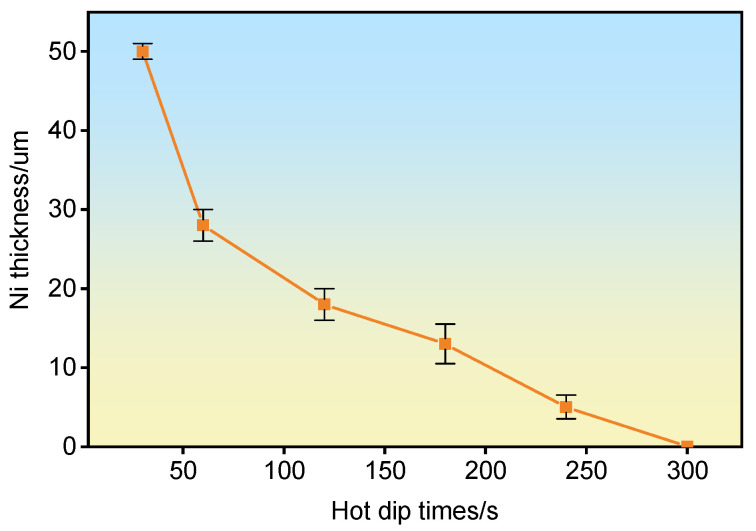
Dissolution curve of nickel-plating layer.

From the line-scan images, it can be seen that Ni elements gradually diffuse into the aluminum matrix with the increase in hot dip time, while there are only traces of Ni elements in the iron matrix. According to the phase diagram, there is only Ni-Fe solid solution near the iron matrix, and Ni elements are easier for Al elements to combine to produce new phases [22]. As the hot immersion time increases, the Ni layer gradually dissolves in the aluminum matrix, and the thickness of the Ni layer shows a gradually decreasing trend. Finally, after 300 s of hot immersion, the Ni layer disappears. Figure 4f is the EDS line scanning result of 300 s of hot dip. It can be seen that the content of Ni element basically tends to zero at this time. No Ni-containing phase was detected at the diffusion layer interface. Only Fe, Si, and Al were found. It is inferred that the kinetic and thermodynamic conditions required for the formation of Al/Ni/Fe ternary metal compounds are not met. Based on the line scan analysis in Figure 4 marking the progressive dissolution thickness of the Ni layer, the dissolution curve of the nickel coating with increasing hot-dip time was plotted as shown in Figure 5.

**Figure 6:**
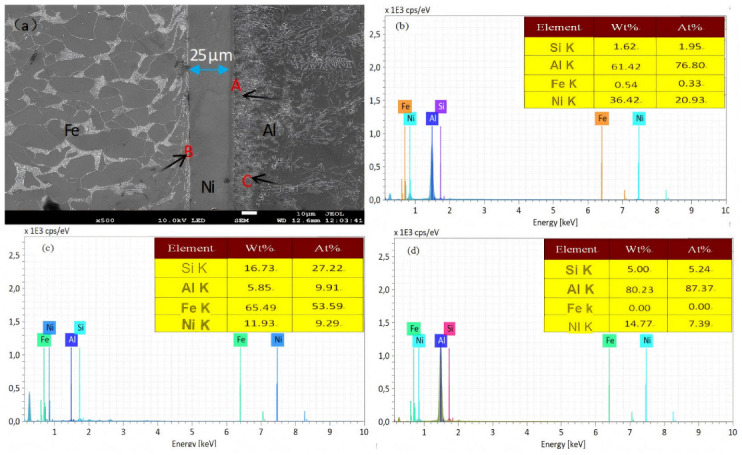
SEM micrographs and compositional analysis of the hot-dip coated specimen (30 s immersion) in as-cast condition. (**a**) Interface organization SEM diagram; (**b**–**d**) present the point-specific EDS compositional analyses corresponding to locations A, B, and C marked in Figure (**a**).

When the hot dip time is 30 s, the interfacial diffusion layer of the nickel-plated steel/Al bimetallic specimen was analyzed for the hot dip 30 s. Figure 6 is the SEM diagram and composition analysis of the hot dip 30 s cast sample.

Figure 6a, it can be seen that the interface consists of Fe matrix, Fe/Ni interface, Ni layer, Al/Ni interface, and aluminum matrix. The thickness of the nickel layer was dissolved from 50 µm of the hot dip sample to about 25 µm, indicating that the dissolution rate of the Ni layer in the hot dip sample was faster than that of the hot dip sample. In Figure 6a, points A and B are the components analysis points of the Al/Ni interface and the Ni/Fe interface, respectively. The components of these two points are summarized in Table 3.

From Figure 6b,c, it can be concluded that the Ni/Al interface generates the NiAl_3_ phase layer. However, the Fe/Ni interface does not generate a phase, but only a Fe-Ni solid solution is generated, but a small amount of Al and SI elements are detected. The aluminum side structure close to the Al/Ni interface is quite different from the silicon phase of the aluminum matrix. Therefore, the composition analysis was performed on point C in Figure 6a, and the analysis showed that the Al-Ni solid solution on the right side of the Al/Ni interface was not detected. Therefore, it is inferred that the Ni layer plays a role in hindering the diffusion of Fe atoms into aluminum.

**Figure 7:**
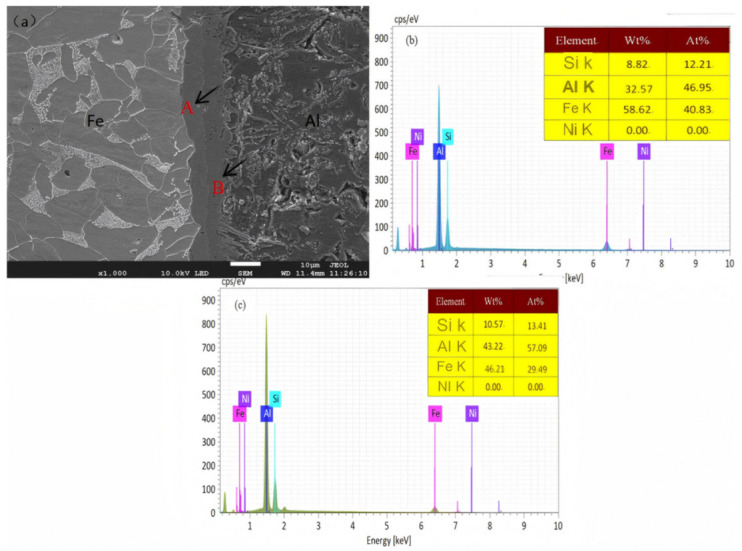
SEM diagram and composition analysis of hot dip 120 s cast specimens. (**a**) Interface organization SEM diagram; (**b**,**c**) point scanning EDS composition analysis.

Figure 7 is the SEM diagram and component analysis of the hot dip 120 s cast sample. The components are analyzed at points A and B in Figure 7a, and the components are summarized in Table 4.

**Figure 8:**
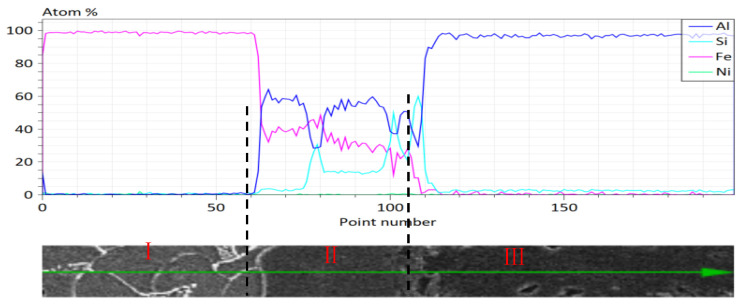
Scanning analysis of cast aluminum/nickel-plated steel wires for hot dip 120 s.

It can be seen that the components of Ni atoms at points A and B are both 0, which means that the diffusion layer does not generate Ni-containing phases, only Al-Fe-Si ternary phases. SPRINGERH et al. [23] conducted a TEM diffraction pattern study on the steel/aluminum diffusion layer, and concluded that the intermetallic phase near the aluminum alloy side was τ_5_–Al_8_Fe_2_Si phase. Position B in Figure 6 is that the atomic ratio of aluminum steel is 1.9:1 near the aluminum alloy side, and the atomic ratio of aluminum steel in the τ_5_–Al_8_Fe_2_Si phase is 4:1, so it is impossible to exist in a single phase. Referring to the Fe-Al phase diagram and looking at related literature, we learned that the FeAl_3_ and Fe_2_Al_5_ phases are the easiest to generate [24,25], and it is inferred that the continuous diffusion layer near the aluminum alloy side consists of τ_5_–Al_8_Fe_2_Si and Fe_2_Al_5_ phases. In Figure 7, the Al/Fe atomic ratio of position A is 1.14:1, while the aluminum steel atomic ratio of the τ_1_-Al_2_Fe_3_Si_3_ phase is 0.7:1. Analysis believes that the diffusion layer close to the steel side coexists with the primary FeAl_3_ phase and the τ_1_-Al_2_Fe_3_Si_3_ phase. In order to intuitively characterize the component distribution of the interface reaction layer, element line scanning analysis was performed on the interface diffusion layer of the aluminum/nickel-plated steel, and the results are shown in Figure 8. The trend of element distribution can be mainly divided into three areas: I, II, and III. Region I is the Fe matrix, mainly Fe element, with the percentage of Fe atoms close to (99%), and there is no Ni element in Region II, and there are mainly three elements, Al, Fe, and Si. The main element in Region III is Al, with a content of nearly 99%.

**Figure 9:**
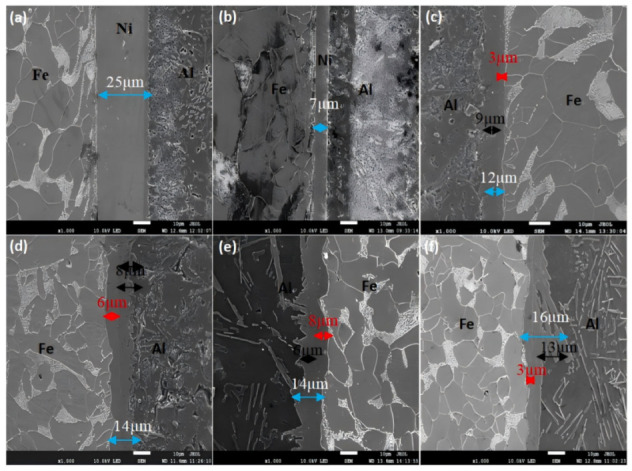
Transition layer thickness of the bimetallic sample interface of nickel-plated steel/Al. Different hot dip times: (**a**) 30 s; (**b**) 60 s; (**c**) 120 s; (**d**) 180 s; (**e**) 240 s; (**f**) 300 s.

Figure 9 illustrates the evolution of transition layer thickness in Ni-coated steel/Al bimetal castings under varying hot-dipping durations. Figure 9a–f correspond to interfacial SEM images of the Ni-coated steel/Al bimetal cast after hot-dipping for 30, 60, 120, 180, 240, and 300 s, respectively. The thicknesses of individual transition layers in Figure 9 were quantified as follows: the τ_5_-Al_8_Fe_2_Si and Fe_2_Al_5_ intermetallic compound (IMC) layers adjacent to the aluminum substrate are marked by black lines, the τ_1_-Al_2_Fe_3_Si_3_ and FeAl_3_ IMC layers near the steel substrate are indicated by red lines, and the average total transition layer thickness is represented by blue lines.

The average thickness of the diffusion layer between aluminum and steel is measured in the figures, as shown in Table 5.

It can be seen that the nickel layer of the sample obtained by hot-dip casting for 30 s and 60 s was not dissolved, and was 25 µm and 7 µm, respectively. However, compared with the thickness of the nickel layer obtained by the sample obtained by hot dip without pouring in Figure 3, the thickness of the sample was greatly reduced. The reason is that after hot dip, the aluminum liquid will not cool down immediately, and it still provides thermal conditions for the dissolution of the nickel layer. The nickel atoms are still diffusing and moving, so the dissolution of nickel-plated steel/Al sample nickel after casting is faster. In Figure 9c, the nickel layer is no longer visible, and an Al-Fe-Si ternary phase layer is generated.

**Figure 10:**
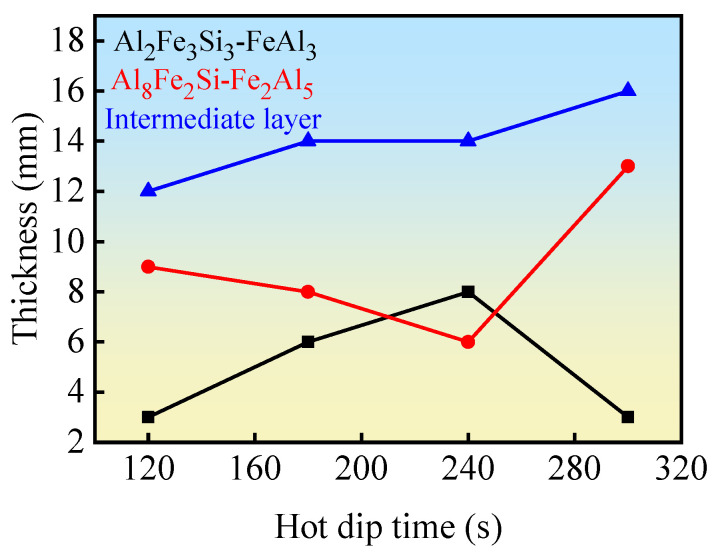
Relationship between the thickness of nickel-plated steel/Al interface diffusion layer and the hot dip time.

According to Figure 9, the average thickness of the intermediate diffusion layer of the aluminum/nickel-plated steel cast at different hot dip times is drawn, as shown in Figure 10.

Because the hot dip is 30 s and 60 s, the nickel layer at the interface has not yet dissolved and no Al/Fe intermetallic compound is generated, so the data of 30 s and 60 s are discarded and the data after hot dip are directly depicted. As can be seen from Figure 10, with the increase in the hot immersion time, the thickness of the τ_1_-Al_2_Fe_3_Si_3_ and FeAl_3_ phase layers increases first and then decreases. During the hot immersion period of 240 s, the thickness of the phase layer reaches the maximum, with a thickness of about 8 µm, while the thickness of the τ_5_–Al_8_Fe_2_Si and Fe_2_Al_5_ phase layers decreases first and then increases, and also reaches the minimum for the hot immersion period of 240 s. The sum of the diffusion layers is gradually increasing, and the thickness of the interfacial diffusion layer of 180 s and 240 s is flat.

### 3.3. Shear Strength

**Figure 11:**
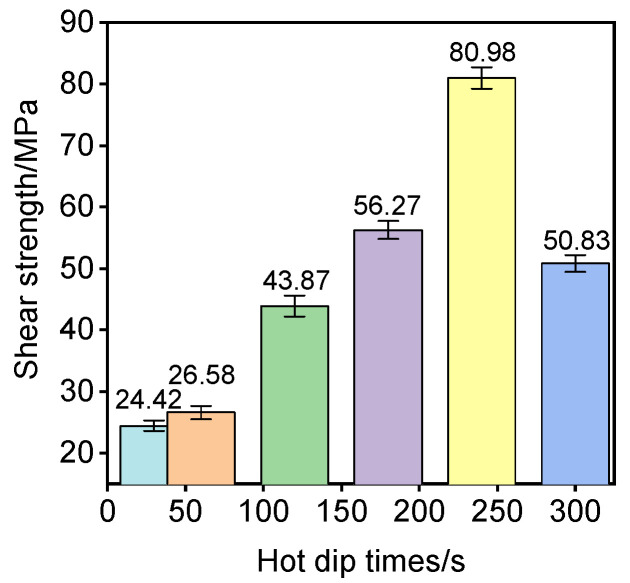
Shear strength after pouring at different hot dip times.

The compression test was carried out using a TYA-600 type electro-hydraulic pressure tester to test the interface shear strength of aluminum/steel separation. The results are shown in Table 6. Figure 11 is a columnar analysis diagram of the shear strength of nickel-plated steel/Al bimetallic specimens after pouring at different hot dip times.

From the hermetic analysis chart of the shear strength, the effect of different hot dip times on the shear strength changes of aluminum/nickel-plated steel bimetallic specimens can be seen. The interface shear strength of the aluminum/nickel-plated steel specimens obtained by hot dip 240 s casting has the highest interface shear strength, and the shear strength reaches 80.98 MPa, which is 75% higher than that of aluminum/steel specimens without an Ni intermediate layer.

## 4. Discussion

### 4.1. Interface Formation Mechanism

When the hot immersion time is 30 s and 60 s, the nickel layer cannot be completely dissolved due to failure to provide sufficient thermodynamic and kinetic conditions. It can be seen from the binary phase diagram of the Al-Ni system that there is a solid solution of Al in Ni in the Al-Ni binary system, and stable metal compounds are also present: Ni_3_Al, Ni_5_Al_3_, NiAl, Ni_2_Al_3_ and NiAl_3_ [26]. The hot dip temperature in the aluminum steel casting process is 730 °C, which exceeds the low melt eutectic temperature of 640 °C. Therefore, there is a white layered flake structure to form an Al-Ni solid solution, and a NiAl phase is precipitated at the interface, and the activated Ni atoms diffuse along the Al grain boundary to the Al matrix to react to form a new NiAl_3_ phase. The NiAl_3_ phase is distributed in the Al matrix in a fine needle-like shape. It can be seen from the Fe-Ni binary phase diagram that there is only the only intermetallic compound FeNi_3_ in the phase diagram, and the others are solid solution tissues [27]. However, the Ni content is relatively different from the Fe component and does not meet the formation conditions. Therefore, the Fe/Ni interface forms a solid solution without stable compounds.

When the hot dip time is 120, 180, 240, and 300 s, the Al/Fe interface formed by the cast nickel-plated steel/aluminum bimetallic samples is formed at this time. At this time, an Al/Fe/Si diffusion layer is generated. Since the melting point (1453 °C) of the Ni element is much higher than the casting temperature (730 °C), diffusion plays a major role in the formation of the interface layer [28]. When the aluminum alloy melt is poured on the surface of nickel-plated steel, under the driving of high temperature, the Ni elements diffuse to the steel side and the Al side, respectively, and the Fe element and Al element also diffuse to the Ni element side. As the temperature drops, the diffusion of the element reaches its limit and then stops diffusion. When the hot immersion time is 120 s, the pure Ni element coating almost completely disappears, indicating that under this process conditions, the 50 μm Ni element coating is completely consumed. Therefore, the Ni element coating with a thickness of 50 μm can block the reaction between Al and Fe. Compared with the unnickel-coated Al/steel samples, it greatly reduces the diffusion of the interface layer and hinders the growth of the brittle compound Fe_2_Al_5_.

### 4.2. Relationship Between Microstructure and Mechanical Properties

When the Ni layer thickness is 50 μm, the presence of the Ni coating successfully affects the formation of interfacial diffusion layer compounds, preventing the formation of Fe_2_Al_5_ IMCs. The continuous diffusion layer formed near the Al side is composed of τ_5_–Al_8_Fe_2_Si and Fe_2_Al_5_ phases, and its thickness is controlled. As we all know, intermetallic compounds with lower hardness at the interface have greater resistance to deformation when subjected to shear forces [29,30] and the hot dip time plays an important role in the bonding strength of nickel-plated steel/aluminum bimetallic interfaces, not because the longer the hot dip time, the better the bonding performance of the interfaces.

Although the Ni layer of the hot dip 30 s and 60 s cast specimens is not completely dissolved, its shear strength is also higher than that of unnickel-plated aluminum/steel bimetals. This also proves that nickel-plated steel can also increase the direct wettability of aluminum/steel bimetals, and at the same time avoid steel oxidation, which is conducive to the combination of aluminum/steel bimetals [31]. When the hot immersion time increases to 120 s, the interface diffusion is insufficient, and Ni atoms preferentially diffuse into Al, which leads to a large thickness of the diffusion layer near the Al side, and the brittle compound Fe_2_Al_5_ is generated, so the interface shear strength is not very high. As the hot immersion time continues to increase, when it increases to 240 s, the interface bonds are best and its shear strength reaches maximum. When the hot dip time exceeds 240 s, Ni atoms are dissolved in the aluminum alloy melt. After casting, the Al/Fe atoms are directly contacted and bound, and the thicker diffusion layer of aluminum/steel bimetal formed by unplating is restored.

Therefore, the ideal interface structure should be composed of IMCs near the Fe side to avoid the formation of brittle compound Fe_2_Al_5_ near the Al side, depending on the process parameters. This principle may also apply to other coated composite castings.

## 5. Conclusions

The present work systematically investigated the effects of hot-dipping duration on the interfacial microstructure and mechanical properties of Ni-coated steel/Al bimetal, leading to the following conclusions:

1. When the thickness of the nickel-plated layer is 50 µm, the hot-dip post-casting process is used to obtain the interface diffusion layer of nickel-plated steel/Al bimetallic. The total thickness of the diffusion layer composed of τ1-Al_2_Fe_3_Si_3_ and FeAl_3_ on the steel side increases first and then decreases. When the hot dip time is 240 s, the thickness reaches the maximum of 8 µm. The diffusion layer composed of τ_5_–Al_8_Fe_2_Si and Fe_2_Al_5_ on the aluminum side decreases first and then increases, and the minimum of 6 µm is reached at 240 s. This confirms hot-dipping time as a critical lever for interfacial architecture control;
2. Peak interfacial shear strength of 80.98 MPa was achieved at 240 s, representing a 75% enhancement over non-Ni-interlayered systems. Strength degradation beyond 240 s correlated with excessive brittle IMC formation and interfacial stress concentration.

Future research directions mainly include two aspects: multi-material interlayer design and process parameter expansion. Explore hybrid interlayers (e.g., Ni/Ag or Ni/graphene nanocomposites) to further suppress detrimental IMC growth while enhancing interfacial ductility. Investigate synergistic effects of hot-dipping temperature (650–750 °C) and pressure (0.1–1.0 MPa) on diffusion kinetics and mechanical interlocking mechanisms.

## Figures and Tables

**Table 1:** Chemical composition of low carban steel (at%).

C	Si	Mn	P	S	Fe
0.2	0.3	0.5	≤0.045	≤0.045	Bal.

**Table 2:** Chemical composition of ZL114 Al alloys (at%).

Si	Fe	Mn	Mg	Ti	Al
7.5	≤0.20	≤0.10	0.45	0.1	Bal.

**Table 3:** Analysis of components of each point of the hot dip 30 s casting sample interface.

Region	Element Contents (at.%)			
	Al	Fe	Si	Ni
A	76.80	0.33	1.95	20.93
B	9.91	53.59	27.22	9.29
C	87.37	0.00	5.24	7.39

**Table 4:** Analysis of components of each point of the hot dip 120 s casting sample interface.

Region	Element Content (at.%)			
	Al	Fe	Si	Ni
A	46.95	40.83	12.21	0.00
B	57.09	29.49	13.41	0.00

**Table 5:** The average thickness of the cast nickel plated steel/Al intermediate diffusion layer at different hot dip times τ.

Hot Dip Time (s)	τ_1_-Al_2_Fe_3_Si_3_ and FeAl_3_ Thickness (μm)	τ_5_-Al_8_Fe_2_Si and Fe_2_Al_5_ Thickness (μm)	Ni Layer Thickness (μm)
30	0	0	25
60	0	0	7
120	3	9	0
180	6	8	0
240	8	6	0
300	3	13	0

**Table 6:** Shear strength after casting at different hot dip times.

Sample	1#	2#	3#	4#	5#	6#
Hot dip time (s)	30	60	120	180	240	300
Shear strength (MPa)	24.42	26.58	43.87	56.27	80.98	50.83

## Data Availability

The original contributions presented in this study are included in the article. Further inquiries can be directed to the corresponding authors.
